# P-1521. Susceptibility of Carbapenem-Resistant Enterobacterales (CRE) and Carbapenem-Resistant *Pseudomonas aeruginosa* (CRPA) With and Without Carbapenemases to Cefepime-Taniborbactam and Comparators: GEARS Antimicrobial Surveillance Program, United States, 2018-2022

**DOI:** 10.1093/ofid/ofae631.1690

**Published:** 2025-01-29

**Authors:** Mark G Wise, Meredith Hackel, Daniel F Sahm

**Affiliations:** IHMA, Schaumburg, Illinois; IHMA, Schaumburg, Illinois; IHMA, Schaumburg, Illinois

## Abstract

**Background:**

Taniborbactam is a novel β-lactamase inhibitor that inhibits serine-β-lactamases and NDM & VIM (but not IMP) metallo-β-lactamases, restoring the activity of cefepime against most isolates of Enterobacterales and *P. aeruginosa* carrying these enzymes. This study examined the *in vitro* activity of cefepime-taniborbactam (FTB) against recent clinical isolates from the US, focusing on genotypically-characterized carbapenem-resistant Enterobacterales (CRE) and carbapenem-resistant *P. aeruginosa* (CRPA).

**Figure:**
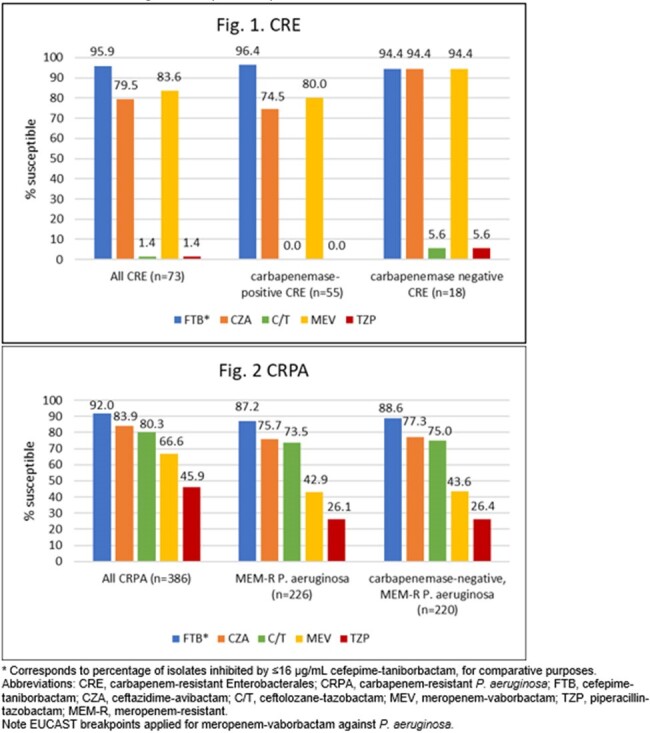

**Methods:**

From 2018-2022, as part of the GEARS program, 4,932 Enterobacterales and 1,508 *P. aeruginosa* isolates were collected from 42 hospitals in the US. MICs of cefepime-taniborbactam and comparators were determined by CLSI reference broth microdilution and interpreted using 2024 CLSI breakpoints. CRE was defined by resistance to meropenem; CRPA was defined by resistance to meropenem and/or imipenem. Isolates with cefepime-taniborbactam MIC ≥16 µg/mL were characterized by whole genome sequencing. Isolates resistant to meropenem were screened for acquired β-lactamases by PCR.

**Results:**

95.9% of the 73 CRE isolates were inhibited by ≤16 µg/mL FTB (Fig. 1). Most CRE (55/73; 75.3%) produced a carbapenemase (40 KPC, 7 NDM, 2 VIM, 2 OXA-48-like, 2 KPC+OXA-48, 1 IMP, and 1 VIM+OXA-48); 96.4% were inhibited by ≤16 µg/mL FTB and the most active comparator was meropenem-vaborbactam (80.0% susceptible). At ≤16 µg/mL, FTB inhibited 92.0% of all CRPA (n=386) and 87.2% of meropenem-resistant CRPA (n=226) (Fig. 2). Among meropenem-resistant CRPA, 6 isolates (2.7%) carried a carbapenemase (2 IMP, 1 IMP+VIM, 1 VIM, 1 GES, 1 NDM). FTB at ≤16 µg/mL inhibited 88.6% of meropenem-resistant, carbapenemase-negative *P. aeruginosa* and 2/3 non-IMP carbapenemase-positive isolates.

**Conclusion:**

FTB at ≤16 µg/mL inhibited >94% of CRE isolates collected in the US, regardless of carbapenemase carriage. Similarly potent activity was observed for cefepime-taniborbactam against meropenem-resistant CRPA isolates, most of which lacked a carbapenemase. Upon approval, cefepime-taniborbactam could be an important option for use against CRE and CRPA, as currently available therapies have limited activity.

**Disclosures:**

**Daniel F. Sahm, PhD**, Pfizer, Inc.: Advisor/Consultant

